# 

**Published:** 1899-03

**Authors:** 


### CONTENTS.

COMMUNICATIONS.

Operations for the Cure of Chronic Empyema of the Antrum........... 101
Are Dentists a Set of Enthusiasts? ........................................ 107
Toothless Twentieth Centenarians, or Vegetarianism—Which? ...... 114
Tooth Forms in Relation to Jaw Movements ............................ 119
Filling Teeth .............................................................. 124
A Method of Handling Alveolar Pyorrhea ................................. 126

PROCEEDINGS.

Ohio State Dental Society, December 8, 1898—Report of the Committee of Tri-State Meeting.............................................. 129

SELECTIONS.

Don't Forget General Wood.................................................. 134
Are We to be a Toothless Race?............................................ 135
Ignorance and Infant Mortality.............................................. 144
Sodium Salicylate for Toothache ........................................... 146
Meager Diet of Porto Rican Peasantry...................................... 146

NOTICES.

American Medical Association................................................ 147
Married ........................................................................ 149
Board of Dental Examiners of Pennsylvania .............................. 149
The British Dental Journal ................................................... 150

OBITUARY.

Emma J. Brophy................................................................ 150

